# Whole Genome Sequencing and Comparative Genomics of Two Nematicidal *Bacillus* Strains Reveals a Wide Range of Possible Virulence Factors

**DOI:** 10.1534/g3.119.400716

**Published:** 2020-01-09

**Authors:** Nik Susič, Sandra Janežič, Maja Rupnik, Barbara Gerič Stare

**Affiliations:** * Agricultural Institute of Slovenia, Plant Protection Department, Ljubljana, Slovenia; and; † Centre for Medical Microbiology, Department for Microbiological Research, National Laboratory of Health, Environment and Food (NLZOH), Maribor, Slovenia

**Keywords:** *Bacillus firmus*, complete genomes, bioinformatics, biological control, nematicidal activity, virulence factors

## Abstract

*Bacillus firmus* nematicidal bacterial strains are used to control plant parasitic nematode infestation of crops in agricultural production. Proteases are presumed to be the primary nematode virulence factors in nematicidal *B. firmus* degrading the nematode cuticle and other organs. We determined and compared the whole genome sequences of two nematicidal strains. Comparative genomics with a particular focus on possible virulence determinants revealed a wider range of possible virulence factors in a *B. firmus* isolate from a commercial bionematicide and a wild type *Bacillus* sp. isolate with nematicidal activity. The resulting 4.6 Mb *B. firmus* I-1582 and 5.3 Mb *Bacillus* sp. ZZV12-4809 genome assemblies contain respectively 18 and 19 homologs to nematode-virulent proteases, two nematode-virulent chitinase homologs in ZZV12-4809 and 28 and 36 secondary metabolite biosynthetic clusters, projected to encode antibiotics, small peptides, toxins and siderophores. The results of this study point to the genetic capability of *B. firmus* and related species for nematode virulence through a range of direct and indirect mechanisms.

*Bacillus firmus* (Bacillales; Bacillaceae) is a species of Gram-positive, rod-shaped bacteria capable of producing endospores under adverse conditions. *B. firmus* is a cosmopolitan species that can be isolated primarily from soil, but also from a variety of different environments including wastewater and marine sediments (Geng *et al.* 2014). *B. firmus* has the potential for biotechnological exploitation, since various strains have been studied for bioremediation applications, such as: enzyme production from agro-industrial waste (Husseiny *et al.* 2008); heavy metals detoxification (Bachate *et al.* 2013; Noroozi *et al.* 2017); textile dye discoloration in wastewater discharge (Rathod and Pathak 2018); and microbial-enhanced heavy oil recovery (Shibulal *et al.* 2018), among others. Importantly, *B. firmus* is an agriculturally useful bacteria, as it has been demonstrated to promote plant growth and alleviate abiotic saline stress (El-Esawi *et al.* 2018), as well as offering plants protection against plant-parasitic nematodes, including root-knot nematodes (RKNs) (Keren-Zur *et al.* 2000; Giannakou *et al.* 2004). RKNs belonging to the *Meloidogyne* genus (Nematoda, Tylenchida, Meloidogynidae) are endoparasites and among the most important plant pests (Sasser 1977; Trudgill and Blok 2001; Bebber *et al.* 2014). RKNs infestations lead to physiologically stressed, low-yielding plants (Abad *et al.* 2003) and 5% crop yield loss on average (Carter and Sasser 1985), significantly contributing to about 110 billion EUR per year in economic damage due to plant-parasitic nematodes in agricultural systems across the world (Danchin *et al.* 2013).

Various broad-spectrum chemical pesticides have been used in crop production for decades to minimize the damage caused by soil-borne plant parasitic nematodes, but many were banned or phased out due to the associated toxicities (Regulation (EC) No. 1107/2009). Biopesticides based on microbial biocontrol agents can be used to control RKNs infestation as a safer alternative to agrochemicals and many candidate bacterial and fungal species have been studied for their potential use as biological nematicides (Wilson and Jackson 2013). Different biopesticides against RKNs are also commercially available, but preparations based on *B. firmus* have seen the widest commercial use - with the *B. firmus* strain I-1582 in the bionematicide preparation BioNem-WP (AgroGreen) being deployed on the market in the early 2000s (Keren-Zur *et al.* 2000; Giannakou *et al.* 2004). Despite commercial use, little is known about the exact mode of action of *B. firmus* against RKNs (Tian *et al.* 2007; Geng *et al.* 2016; Valencia and Kotcon 2016). Bacterial proteases are considered to be the primary virulence factor in nematicidal *B. firmus* strains (Lian *et al.* 2007; Tian *et al.* 2007; Geng *et al.* 2016). However, *Bacillus* spp. are known to produce various secondary metabolites, enzymes and toxins, which have never been studied for nematicidal activity. One example is a cereulide-like emetic toxin purified from *B. firmus* ATCC 14575^T^ and ATCC 8247 strains (Taylor *et al.* 2005). Additionally, *Bacillus* spp. may inhibit RKNs or alleviate their effects on plants indirectly by strengthening plant defense mechanisms (Kloepper *et al.* 2004), release of repellents (Valencia and Kotcon 2016), and plant growth-promotion (El-Esawi *et al.* 2018). Screening for secondary metabolite production potential in nematicidal bacteria could thus be warranted since there are various secondary metabolite compounds from other organisms with substantial nematotoxic properties (Khalil 2013). With the accessibility of next-generation sequencing, bacterial genomes can be assessed through bioinformatics analysis for the genetic potential to produce nematicidal substances, including novel secondary metabolites and virulence factors useful in agriculture. This approach has been used by Zheng *et al.* (2016) to screen the genomic sequences of 120 *Bacillus* strains exhibiting nematicidal activity against bacterivorous nematode *Caenorhabditis elegans* for the presence of various virulence factors. The whole-genome sequence analysis of *B. firmus* strain DS-1 was also the first step in the determination of nematicidal serine protease Sep1, capable of inhibiting the growth and development of the nematodes *C. elegans* and *Meloidogyne incognita* (Geng *et al.* 2014; 2016). Bioinformatics investigation of the nature of *B. firmus* virulence against plant parasitic nematodes like RKNs is however still lacking – especially assessment of the range of possible virulence factors that could be found in *B. firmus* genomes.

The aim of this study was to compare the range of possible virulence determinants in the genomes of two geographically and phylogenetically distinct nematicidal *Bacillus* strains – a *B. firmus* isolate from widely used commercial bionematicide and a local wild-type *Bacillus* sp. isolate with nematicidal activity.

## Materials and Methods

### Bacterial strains

Two *Bacillus* strains with nematicidal potential were used in this study. Strain *B. firmus* I-1582 was isolated from the commercially available biological seed treatment preparation VOTiVO FS (Bayer CropScience, Germany), which is used to protect against soil-borne plant parasitic nematodes. Strain *Bacillus* sp. ZZV12-4809 was isolated from the pea (*Pisum sativum* L.) rhizosphere, in Maribor, Slovenia. Both strains were cultivated in LB liquid and solid media at 23°. The nematicidal activity of both isolates has been demonstrated in prior *in vitro* and pot experiments (Susič *et al.* 2019). For endospore differential staining, bacterial cultures in exponential growth phase were inoculated into liquid Difco Sporulation Medium (DSM) (Monteiro *et al.* 2014) for 48h at 37°, stained according to Schaeffer and Fulton (1933) and visualized under a microscope.

### Species identification

For bacterial identification, total bacterial DNA was isolated from a single bacterial colony grown on an LB agar plate at 23° for 48h. Bacteria were identified according to the 16S rRNA gene sequence, which was amplified using the forward 27f (5′-GAGAGTTTGATCCTGGCTCAG-3′) and reverse 1495r (5′-CTACGGCTACCTTGTTACGA-3′) primers and with cycling conditions as described by Bandi *et al.* (1994). 16S rRNA sequences were first searched against quality-controlled databases of 16S rRNA sequences in the EzBioCloud Identify service (Yoon *et al.* 2017). Second, the average nucleotide identity (ANI) was calculated based on the genomes of *B. firmus* I-1582, *Bacillus* sp. ZZV12-4809 and five other *B. firmus* sequences available in GenBank: *B. firmus* DS1 (GenBank assembly accession: GCA_000565285.1), *B. firmus* LK28 (GCA_001038755.1), *B. firmus* NBRC 15306 (GCA_001591465.1), *B. firmus* NCTC10335 (GCA_900445365.1), *B. firmus* 14_TX (GCA_003315495.1) and *B. oceanisediminis* 2691 (GCA_000294775.2), using JSpeciesWS (Richter *et al.* 2016; http://jspecies.ribohost.com/jspeciesws/#home), implementing the algorithms as described by Goris *et al.* (2007). JSpeciesWS was also used to calculate tetra-nucleotide signatures (Tetra) correlation indexes and to search the associated database for related genomes using the Tetra Correlation Search (TCS) function. For general phylogenetic positioning of *B. firmus* I-1582 and *Bacillus* sp. ZZV12-4809 within the genus, we included the two genomes in phylogenetic analysis of 222 assembled *Bacillus* genomes from type material available in GenBank. Phylogenetic analysis was carried out with GToTree v1.1.10 (Lee 2019; https://github.com/AstrobioMike/GToTree), using the hidden Markov model (HMM) single-copy gene set (119 HMMs) for Firmicutes and the maximum likelihood (ML) method for tree reconstruction. The best-fitting substitution model was selected according to the Akaike information criterion (AIC) and Bayesian information criterion (BIC) scores, with consensus trees calculated from 1000 bootstrap replicates. Heatmaps visualizing the ANI values (%) and phylogenetic trees were rendered in R with the packages ‘Superheat’ (Barter and Yu 2018), ‘ggtree’ (Yu *et al.* 2017) and ‘ggplot2’ (Wickham 2016).

### Genome sequencing, assembly and annotation

For sequencing, total DNA was isolated from overnight cultures of *B. firmus* I-1582 and *Bacillus* sp. ZZV12-4809 with the QIAamp DNA Mini Kit (Qiagen, Germany). Paired-end libraries were prepared following the manufacturer’s instructions with the Nextera XT DNA Library Preparation Kit (Illumina, USA) and then sequenced on a MiSeq System (Illumina) using the MiSeq Reagent Kit v3 (600-cycle) (Illumina). Raw sequence reads were adapter-trimmed (Trimmomatic) and quality filtered (FastQC). Genome sequences were assembled *de novo* and annotated using the Comprehensive Genome Analysis Service at the Pathosystems Resource Integration Center (PATRIC, https://www.patricbrc.org) bioinformatics utility (Wattam *et al.* 2017). The assembly and annotation pipeline was optimized for Illumina MiSeq reads and involved a Velvet (Zerbino and Birney 2008) run with hash length 35, error correction of sequenced reads with BayesHammer (Nikolenko *et al.* 2013), and assembly with SPAdes (Bankevich *et al.* 2012), with a k-mer value of up to 99. Assembly Rapid Annotation using Subsystem Technology (ARAST) quality score was used to sort the assembly results. Assemblies were additionally assessed with BlobTools (Laetsch and Blaxter 2017) to detect contaminant DNA sequences. Genome assemblies were annotated with the RAST tool kit (RASTtk) (Brettin *et al.* 2015). The annotation included functional assignments of predicted protein coding sequences (CDS) to Enzyme Commission (EC) numbers (Schomburg *et al.* 2004), Gene Ontology (GO) assignments (Ashburner *et al.* 2000), and mapping to KEGG pathways (Kanehisa *et al.* 2016), genus-specific protein families (PLFams) and cross-genus protein families (PGFams) (Davis *et al.* 2016). Additionally, predicted protein sequences were separately assigned to clusters of orthologous groups (COG) using eggNOG-mapper v4.5.1 (Huerta-Cepas *et al.* 2017). Genome assemblies and syntenic regions were visualized as circular maps using plotMyGBK (https://github.com/microgenomics/plotMyGBK) and GGisy (https://github.com/Sanrrone/GGisy) python scripts that depend on the R environment (R Core Team 2018), with the packages ‘data.table’ (Dowle *et al.* 2019), ‘Rsamtools’ (Morgan *et al.* 2019) and ‘OmicCircos’ (Hu *et al.* 2014). Where applicable, various graphic elements were arranged with Inkscape v0.92.4 (https://inkscape.org).

### Comparative genomics and bioinformatics analysis

The OrthoVenn web server with default parameters (E-value 1e−5 and inflation value 1.5) (Wang *et al.* 2015; http://www.bioinfogenome.net/OrthoVenn/) was used to identify orthologous gene clusters in the assembled genomes of *B. firmus* I-1582 and *Bacillus* sp. ZZV12-4809, and five other *B. firmus* sequences available in GenBank as described above. Homologs to known transporters, virulence factors, drug targets and antibiotic resistance genes, were identified using PATRIC by homology to known sequences in the following databases: Transporter Classification Database (TCDB) (Saier *et al.* 2016), Virulence Factor Database (VFDB) (Chen *et al.* 2016), PATRIC_VF (Mao *et al.* 2015), Therapeutic Target Database (TTD) (Zhu *et al.* 2010), DrugBank 4.0 (Law *et al.* 2014), Comprehensive Antibiotic Resistance Database (CARD) (McArthur *et al.* 2013) and the National Database of Antibiotic Resistant Organisms (NDARO). Antimicrobial resistance (AMR) genes were also annotated by using the k-mer-based AMR gene detection method, which utilizes PATRIC’s curated collection of representative AMR gene sequence variants (Wattam *et al.* 2017). Putative nematode-virulent proteases were revealed by protein BLAST search on the assembled genomes, by sequence similarity with an e-value of 10^−5^ and minimum 30% sequence identity over 60% of both protein lengths (Rost 1999; Galagan *et al.* 2003). Selected protein sequences were aligned with Clustal Omega (https://www.ebi.ac.uk/Tools/msa/clustalo/) to calculate the percent (%) identity matrix and presented in a heatmap. The annotated genome assembly sequence files, including information for contigs and ORFs, were analyzed with antiSMASH v4.2.0 (Blin *et al.* 2017) and RIPPMiner with pairwise BLAST (Agrawal *et al.* 2017) to predict and analyze various types of secondary metabolite gene clusters.

### Data availability

The complete genome sequence data, including raw sequence reads, genome assemblies and annotations of *B. firmus* I-1582 and *Bacillus* sp. ZZV12-4809 used in this study were submitted to NCBI, GenBank under the BioProject accession ID: PRJNA533096. Supplemental material available at figshare: https://doi.org/10.25387/g3.11522544.

## Results and Discussion

### Microbial features and identification of bacterial species

**Figure 1:**
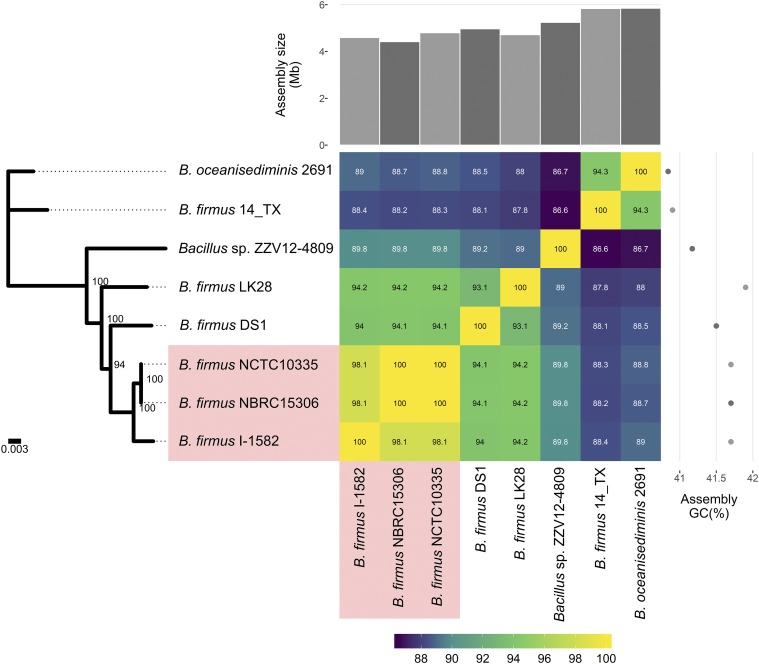
Maximum likelihood phylogenetic tree based on the 8 available genomic assemblies of *Bacillus firmus* and *Bacillus oceanisediminis* 2691 with heatmap annotation. Nodes annotated with bootstrap support. Average nucleotide identity (ANI) values (%) are presented in a heatmap, ranging from lower (violet) to higher sequence identity (green-yellow), and clustered according to the phylogenetic tree. ANI analysis showed 100% sequence identity between *B. firmus* NBRC 15306 and *B. firmus* NCTC 10335, which is in accordance with information from the NCBI GenBank (data not shown). *B. firmus* I-1582 exhibited 98.14% sequence identity with strains NBRC 15306 and NCTC 10335 (highlighted in red), and 89.83% sequence identity with *Bacillus* sp. ZZV12-4809. The heatmap was annotated with a bar chart showing the varying sizes (Mb) of all 8 assemblies (at the top) and their respective GC (%) content (right-hand side).

Colony and cell morphology as well as 16S rRNA gene and whole genome sequence analysis were performed to determine species identity of the two studied nematicidal bacterial strains. Strains I-1582 and ZZV12-4809 generally formed round, pale-cream colonies on LB agar medium under the described growth conditions, with 2 µm-long rod-shaped cells able to form endospores (Supplementary Figure 1B,C). *B. firmus* I-1582 and *Bacillus* sp. ZZV12-4809 16S rRNA gene sequence amplification (Supplementary Figure 1A) and EzBioCloud analysis showed 100% and 99.23% similarity (Supplementary Table 1), respectively, to the 16S rRNA sequence of *B. firmus* strain NBRC 15306^T^ (GenBank accession: BCUY01000205). Tetra analysis was in accordance with 16S rRNA results, and a TCS search for related genomes returned *B. firmus* DS1 as the closest genome in the database to I-1582 and ZZV12-4809, with Z-scores of 0.99866 and 0.99709, respectively (Supplementary Table 2B,C). Average nucleotide identity values (ANI %) were calculated between *B. firmus* I-1582, *Bacillus* sp. ZZV12-4809 and all 5 *B. firmus* genome assemblies currently publicly available in GenBank, with *Bacillus oceanisediminis* 2691 as an outgroup (see Materials and Methods). I-1582 and ZZV12-4809 shared moderate to high (ANIb/ANIm) identity with each other, as well as with the type *B. firmus* strain NBRC 15306 (Figure 1; Supplementary Table 2). According to the strict 95% cut-off value to delineate species boundary (Richter and Rosselló-Móra 2009; Chun *et al.* 2018), strain I-1582, but not ZZV12-4809, could be considered as *B. firmus*. Phylogenetic analysis within the genus *Bacillus* to discern the phylogenetic position of this strain accompanied by the ANI values showed that ZZV12-4089 was still most closely related to the *B. firmus* group (Supplementary Figure 2). ANI calculations and phylogenetic analysis indicated previously unreported variability within the strains of *B. firmus* that points to incorrect species circumscription in some strains. Therefore, description of a novel species could be warranted (Figure 1).

### B. firmus I-1582 and Bacillus sp. ZZV12-4809 share general genome characteristics

The genome assemblies of *B. firmus* I-1582 and *Bacillus* sp. ZZV12-4809 were 4,597,711 and 5,245,841 bp long, and yielded 199 and 90 contigs, respectively (Table 1; Supplementary Figures 3,4). The genomes of the two strains have slightly different G+C content and similar numbers of tRNA and rRNA genes. In *B. firmus* I-1582, 75.4% of predicted coding sequences (CDSs) were assigned to the clusters of orthologous groups (COG) categories, and in *Bacillus* sp. ZZV12-4809 74% of predicted CDSs were assigned to COGs, although the functional category of many CDS could not be defined (Table 2). Multiple syntenic blocks were found to be shared with *B. firmus* I-1582 and *Bacillus* sp. ZZV12-4809 assemblies. High-synteny blocks (above 90% nucleotide identity), equal to or larger than 15 kbp, were present in 11 contigs in ZZV12-4809, aligning to 15 contigs in I-1582 (Supplementary Figure 5). Gene assignment to the COG categories indicated similar fractions of CDS assigned to different categories in both strains. Some differences in COG categories were observed between I-1582 and ZZV12-4809; certain categories contain a larger fraction of genes in the first while others in the second corresponding genomes (Table 2).

**Table 1:** Sequencing, assembly and annotation information for the *Bacillus firmus* I-1582 and *Bacillus* sp. ZZV12-4809 genomes

	*B. firmus* I-1582	*Bacillus* sp. ZZV12-4809
Assembly information		
Assembly size (bp)	4,597,711	5,245,841
Number of contigs	199	90
G+C content (%)	41.70	41.17
Largest contig (bp)	277,315	587,391
N50 (bp)	55,490	172,048
L50	24	9
Estimated coverage (times ×)	158	58
**Annotation information**		
CDS	5,048	5,671
rRNA genes	23	15
tRNA genes	103	102
Hypothetical proteins	1,600	1,953
Proteins with functional assignments	3,448	3,718
Proteins with EC number assignments	1,110	1,135
Proteins with GO assignments	934	957
Proteins with Pathway assignments	848	863
Proteins with PATRIC genus-specific family (PLfam) assignments	4,498	4,562
Proteins with PATRIC cross-genus family (PGfam) assignments	4,518	4,611

**Table 2:** The number and percentage (%) of genes assigned to COG categories in the genomes of *Bacillus firmus* I-1582 and *Bacillus* sp. ZZV12-4809

	*B. firmus* I-1582		*Bacillus* sp. ZZV12-4809	
	Gene number	Gene percent (%)	Gene number	Gene percent (%)
CDS assigned to COG categories	3,805	100	4,194	100
**COG category**				
[n] Not assigned to any COG category	365	8.75	424	9.18
[m] Assigned to multiple COG categories	55	1.32	58	1.26
[B] Chromatin structure and dynamics	1	0.02	1	0.02
[C] Energy production and conversion	230	5.52	224	4.85
[D] Cell cycle control, cell division, chromosome partitioning	39	0.94	36	0.78
[E] Amino acid transport and metabolism	340	8.15	381	8.25
[F] Nucleotide transport and metabolism	88	2.11	94	2.04
[G] Carbohydrate transport and metabolism	189	4.53	245	5.31
[H] Coenzyme transport and metabolism	130	3.12	113	2.45
[I] Lipid transport and metabolism	118	2.83	124	2.69
[J] Translation, ribosomal structure and biogenesis	172	4.13	174	3.77
[K] Transcription	253	6.07	306	6.63
[L] Replication, recombination and repair	239	5.73	223	4.83
[M] Cell wall / membrane / envelope biogenesis	175	4.20	183	3.96
[N] Cell motility	40	0.96	38	0.82
[O] Posttranslational modification, protein turnover, chaperones	113	2.71	121	2.62
[P] Inorganic ion transport and metabolism	217	5.20	254	5.50
[Q] Secondary metabolites biosynthesis, transport and catabolism	55	1.32	69	1.49
[S] Function unknown	1081	25.92	1257	27.22
[T] Signal transduction mechanisms	163	3.91	174	3.77
[U] Intracellular trafficking, secretion, and vesicular transport	29	0.70	31	0.67
[V] Defense mechanisms	78	1.87	88	1.91

### Comparative genomics reveals high overall similarity and certain strain-specific gene clusters

**Figure 2:**
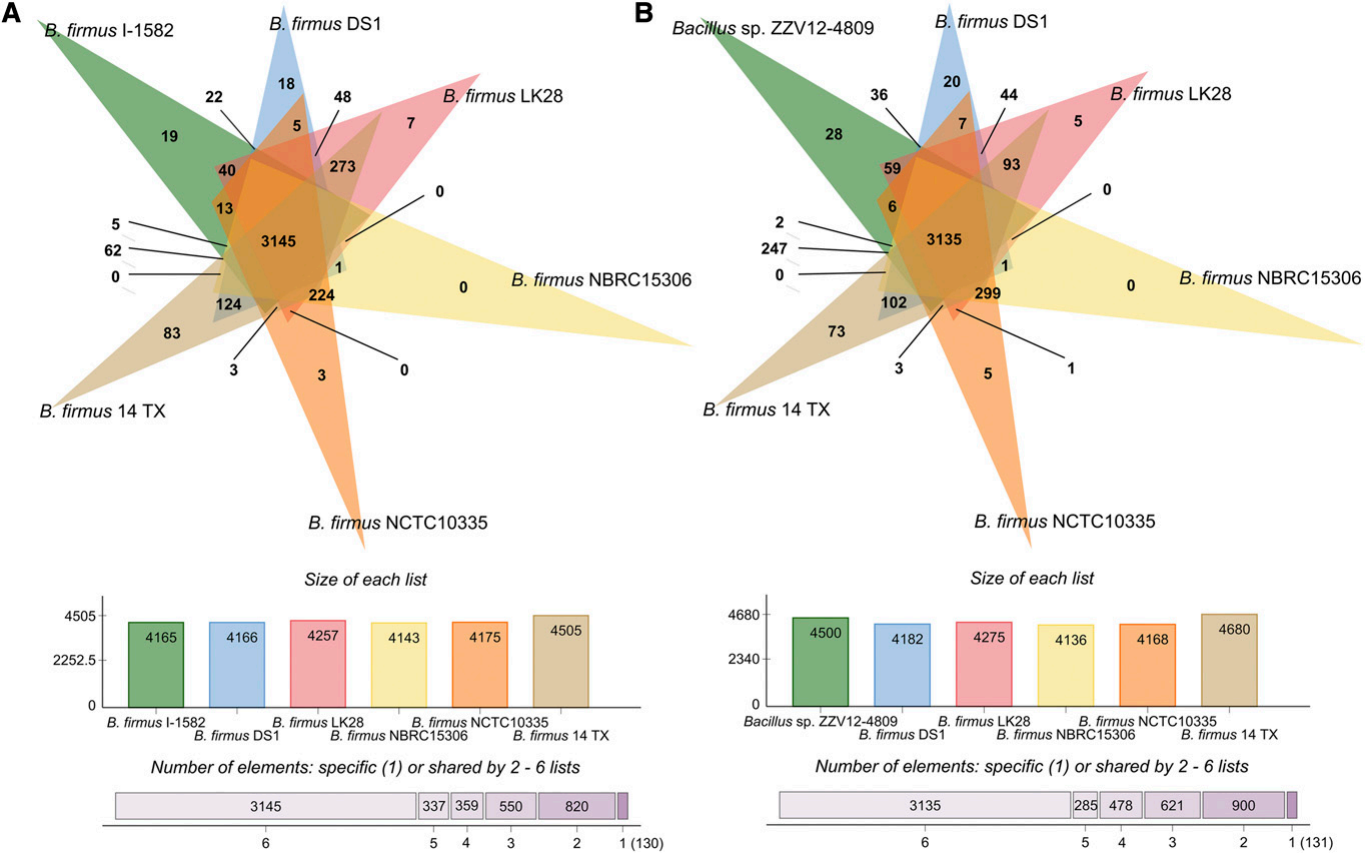
Venn diagrams generated by OrthoVenn showing orthologous clusters shared separately by A) *Bacillus firmus* I-1582 (green) and B) *Bacillus* sp. ZZV12-4809 (green) and five other *Bacillus* species, specifically *B. firmus* DS1 (blue), LK28 (red), NBRC 15306 (yellow), NCTC10335 (orange), and 14_TX (brown). When A) *B. firmus* I-1582 was compared with other *B. firmus* strains, there were 820, 550, 359, 337 and 3145 clusters shared between 2, 3, 4, 5 and 6 strains, respectively, while 130 clusters (clusters of singleton genes) were found in only 1 of the 6 *Bacillus* strains compared. Comparison of B) ZZV12-4809 with other strains resulted in 900, 621, 478, 285 and 3135 clusters shared between 2, 3, 4, 5 and 6 strains, respectively, while 131 clusters of singleton genes were found in only 1 of the 6 strains compared.

Genome-wide analysis of orthologous clusters is frequently used in comparative genomics studies – commonly in whole genome analysis across species, as orthologous genes are clusters of genes in different species, originating by vertical descent from a single gene in the last common ancestor (Wang *et al.* 2015). Genome-wide analysis of orthologous clusters revealed that the I-1582 and ZZV12-4809 genomes shared the vast majority of gene families / orthologous clusters (3850), while there were 1.03 and 2.33% specific clusters in I-1582 and ZZV12-4809 cluster lists, respectively. Expanding on the ANI results, the I-1582 and ZZV12-4809 proteomes were separately compared with five other *B. firmus* in the GenBank database (Figure 2). The strains formed 5341 clusters, 5211 orthologous clusters (listed in at least two strains) and 3116 single-copy gene clusters, when I-1582 was compared with other strains (Figure 2A); or 5550 clusters, 5419 orthologous clusters and 3096 single-copy gene clusters when ZZV12-4809 was compared with others (Figure 2B). Apart from the orthologous clusters shared by all the strains, some strains shared a proportionally higher number of clusters with each other. 14_TX shared almost 4 times more additional orthologous clusters with ZZV12-4809 than with I-1582, despite the fact that 14_TX and I-1582 share a higher nucleotide identity. This result could be due to the fact that there were simply more clusters formed by the ZZV12-4809 list than I-1582. Comparison of I-1582 and ZZV12-4809 lists identified 40 and 92 clusters respectively specific to I-1582 or ZZV12-4809. These strain-specific clusters could potentially be involved in important biological processes, so affiliations across GO terms were checked for differences between the strains. Genes in these clusters seemed to be involved in different molecular functions in I-1582 or ZZV12-4809 (Supplementary Table 4).

### Antimicrobial resistance genes, drug targets, transporters and virulence factors

Groups of genes that could contribute toward virulent activity of studied bacterial strains were investigated. The *B. firmus* I-1582 and *Bacillus* sp. ZZV12-4809 genomes were respectively annotated with 75 and 87 genes homologous to known transporters, virulence factors, drug targets and antibiotic resistance genes (Table 3). The two genomes shared more than 80% of these genes. Most of the shared genes were associated with resistance to triclosan, phenicol, peptide, aminoglycoside and tetracycline classes of antibiotics. However, strain ZZV12-4809 possessed 14 unique genes not present in I-1582 (Supplementary Table 5) – most of which seemed to be involved in resistance to various antibiotics. Among these, there were genes *vgbA* and *vatD* involved in resistance to streptogramin antibiotics; *bcrC*, bacitracin resistance; *vanR*, glycopeptide antibiotic resistance; *fosB*, fosfomycin resistance; *catA9*, resistance to chloramphenicol; *rlmAII*, resistance to the macrolide antibiotics tylosin and telithromycin (Takaya *et al.* 2013); and genes *blaA* and *blaD*, involved in resistance to β-lactam antibiotics. Of the genes present in strain ZZV12-4809 but not in strain I-1582, there were also *capB* and *capC*, which were classified as virulence factors involved in anti-phagocytosis (Candela and Fouet 2006); and *aapA* encoding an Amino-acid permease AapA transporter. Genes for putative gap-family peptidoglycolipid transporter and *hemH* for coproporphyrin ferrochelatase (EC 4.99.1.9) were found in I-1582 but not in ZZV12-4809. The latter gene product was classified as a drug target for N-Methylmesoporphyrin (Supplementary Table 5).

**Table 3:** Number of genes associated with antimicrobial resistance, drug targets, transporters, various virulence factors and secondary metabolites found in *Bacillus firmus* I-1582 and *Bacillus* sp. ZZV12-4809 genome assemblies

	Number of determinants in the genome	
Associated process	*B. firmus* I-1582	*Bacillus* sp. ZZV12-4809
Antimicrobial resistance genes	40	48
Drug targets	12	11
Transporters	13	13
Human / animal virulence factors	6	9
**Nematode virulence factors**		
Proteases	18	19
Chitinases	0	2
Secondary metabolite clusters	28	36

### Multiple nematode virulence factors in I-1582 and ZZV12-4809 genomes

In order to assess the virulence potential of the two sequenced *Bacillus* strains, we performed a BLASTP search of the previously described virulence factors of bacterial, fungal, plant and animal origin surveyed by Zheng *et al.* (2016), bacterial proteases identified by Geng *et al.* (2016), and whole-genome biosynthetic cluster prediction. The candidate virulence factors found were classified into classes such as proteases (Supplementary Table 6), chitinases (Supplementary Table 8), peptides, and other secondary metabolites – such as putative siderophore compounds and toxins (Supplementary Table 9).

#### Proteases:

**Figure 3:**
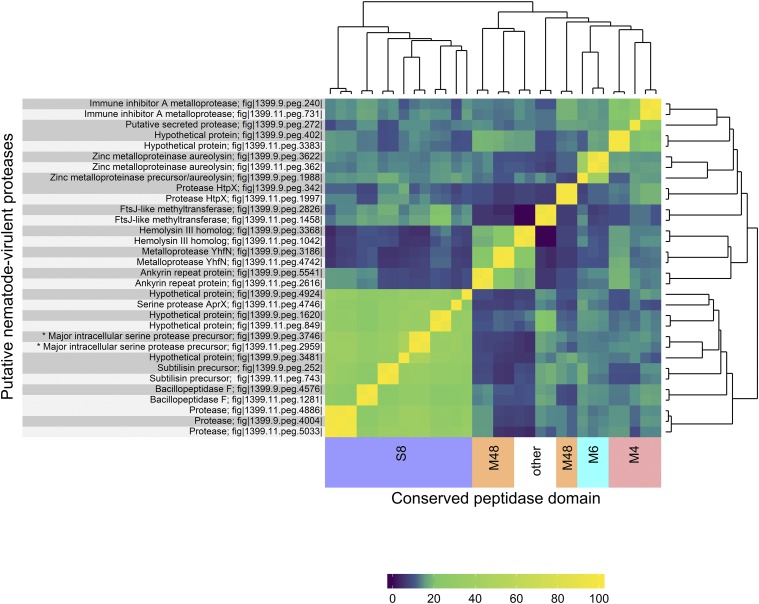
Clustering of putative nematode-virulent proteases found in *Bacillus firmus* I-1582 (light-gray) and *Bacillus* sp. ZZV12-4809 (dark-gray) genomes. All-against-all comparison of amino-acid sequences is based on percent (%) identity values calculated with Clustal Omega and presented in a heatmap, ranging from lower (violet) to higher sequence identity (green-yellow). A dendrogram was created using hierarchical clustering by Euclidean distance. The identities of analyzed proteins are given as genomic annotations together with PATRIC IDs (left-hand margin). Colored highlights (bottom margin) represent clustering of sequences according to the conserved peptidase family domains: S8 (blue), M4 (magenta), M6 (cyan), M48 (orange), or other (no color). Asterisks (*) denote high-similarity protein sequences to the nematicidal protease Sep1 from *B. firmus* DS1.

Using known nematode-virulent protease sequences as queries (Supplementary Table 6), respectively 18 and 19 different homologous sequences were found in *B. firmus* I-1582 and *Bacillus* sp. ZZV12-4809 (Supplementary Table 7). The sequences clustered loosely into groups based on the conserved family domains, as determined with InterProScan. Most of them belonged to the peptidase S8 domain family (Figure 3). Five, including Immune inhibitor A metalloproteases (InhA), hypothetical proteins and one putative secreted protease found only in ZZV12-4809, belonged to the M6 peptidase family. Three sequences annotated as Zn-metalloproteinase aureolysin (EC 3.4.24.29), were found to have conserved peptidase M4 domain. Six protein sequences were found to contain M48 peptidase domains, although two of these, annotated as HtpX proteases, had substantially shorter amino-acid sequences and did not cluster with the rest of the group. Haemolysin III homologs clustered more readily with peptidase M48 family proteins, while haemolysin A homologs clustered better with peptidase S8 family proteins. Homologs of haemolysin A in *B. firmus* genomes had similar domains to TlyA from *Mycobacterium tuberculosis*, which is known to exhibit hemolytic exotoxin activity *in vitro* (Rahman *et al.* 2010). Haemolysin III homologs from I-1582 and ZZV12-4809 showed high sequence identity with the haemolysin III family protein from *B. firmus* DS1, which exhibited some nematicidal activity *in vitro* against *C. elegans* N2 (Geng *et al.* 2016). Most of the putative nematode-virulent proteases had direct homologous counterparts in either the I-1582 or ZZV12-4809 genome, as indicated by high identity correlation in amino-acid sequences (Figure 3). One protease gene ‘fig|1399.11.peg.4746’ in the I-1582 did not have a counterpart in the ZZV12-4809 genome while 4 genes (‘fig|1399.9.peg.4924’, ‘fig|1399.9.peg.3481’, ‘fig|1399.9.peg.1988’ and ‘fig|1399.9.peg.272’) in ZZV12-4809 did not have a counterpart in the I-1582. This study revealed numerous putative nematode-virulent proteases in the *B. firmus* I-1582 and *Bacillus* sp. ZZV12-4809 genomes. However, previous work with *B. firmus* DS1 has demonstrated that most of the tested putative proteases did not exhibit any nematicidal effects and only recombinant protease EWG10090 (Serine protease 1; Sep1) led to significant 73.2% mortality of *C. elegans* N2 when expressed (Geng *et al.* 2016). Homologs to Sep1 were present in the *B. firmus* I-1582 and *Bacillus* sp. ZZV12-4809 genomes. Protein sequences ‘fig|1399.11.peg.2959’ in I-1582 and ‘fig|1399.9.peg.3746’ in ZZV12-4809 showed 98% and 93% identity respectively with Sep1 from *B. firmus* DS1.

#### Chitinases:

Chitinases are considered to be putative nematode-virulence factors, since they may digest chitinous components of nematode eggs. Additionally, chitinases can enhance the deleterious effects of proteases, as postulated previously. Zheng *et al.* (2016) found that *Bacillus* strains with genes encoding putative virulent proteases and chitinases had higher nematode mortality rates when compared to strains with only protease genes. Through an initial BLAST search, two putative chitinase sequences were found only in the *Bacillus* sp. ZZV12-4809 genome, but were below the selection threshold (see Materials and Methods). Upon searching the NCBI Conserved Domain Database (CDD), both protein sequences were found to contain the chitin-hydrolyzing GH18-chitinase domain and could thus be considered putative nematode-virulent chitinases. Sequence ‘fig|1399.9.peg.2658’ showed the highest sequence identity with chitinase ABW96521.1 (33%; E-value = 1.0e-31) from the fungus *Pochonia chlamydosporia*, while sequence ‘fig|1399.9.peg.3724’ showed highest sequence identity with chitinase ABP37997.1 (26%; E-value = 1.0e-17) from the fungus *Purpureocillium lilacinum*. Both fungi are nematophagous and have been shown to parasitize eggs of the RKN *Meloidogyne enterolobii* (Silva *et al.* 2017). Through a recursive BLAST search, two additional sequences were found in the I-1582 assembly and one in ZZV12-4809 but appeared to lack the catalytic chitinase domain (Supplementary Table 8).

#### Secondary metabolites:

The secondary-metabolite gene clusters (antiSMASH) analysis revealed the presence of 28 and 36 predicted clusters in strains I-1582 and ZZV12-4809, respectively. Of these, only 9 and 12 were assigned to specific types, and five predicted clusters in each genome assembly (clusters 11, 21, 24, 25 and 28 in strain I-1582, and clusters 10, 11, 16, 17 and 35 in strain ZZV12-4809) were found on the contig borders and were likely to be incomplete (Supplementary Table 9). One gene cluster in strain I-1582 and two gene clusters in the ZZV12-4809 genome were predicted to be involved in the synthesis of the siderophore petrobactin which is considered a virulence factor of *Bacillus anthracis* (Wilson *et al.* 2010) and is the primary siderophore produced by this bacterium under iron starvation (Koppisch *et al.* 2005). Due to the biological importance of iron (Beneduzi *et al.* 2012), siderophore production by rhizobacteria can indirectly inhibit the growth of phytopathogenic soil microorganisms by limiting the bioavailability of iron, or promote plant growth through plant-microbe interactions (Singh *et al.* 2011). In I-1582, one lanthipeptide was predicted with 43.5% sequence identity (e = 5e-04) with paenicidin A. Two lanthipeptides were predicted in ZZV12-4809, the first showed very weak sequence identity with plantazolicin (44%, e = 2.9) and the second showed 38.9% identity (e = 6e-10) with cerecidin A7. *Bacillus* spp. are known to produce class I and II lanthipeptides with antibacterial activity – lantibiotics (Barbosa *et al.* 2015). Clusters 10 and 24 in the ZZV12-4809 genome were found to contain genes associated with bacteriocin-terpene (cluster 10) or bacteriocin (cluster 24) synthesis, while in I-1582, one cluster associated with terpene synthesis was predicted. Bacteriocins are antimicrobial peptides produced by bacteria, which have the ability to kill or inhibit closely related bacterial strains without the negative effects on bacteriocin-producing bacterium (Yang *et al.* 2014). In ZZV12-4809, cluster 19 was predicted to contain a hybrid type I polyketide synthase-nonribosomal peptide synthetase (PKS-NRPS) system, with 35% of genes similar to paenilamicin biosynthetic cluster. Paenilamicin has previously been identified as an antibiotic compound produced by the honey bee pathogen *Paenibacillus larvae* (Garcia-Gonzalez *et al.* 2014). Additionally, polysaccharide-type cluster 5 in I-1582 was found to contain two genes similar to *aveBIII* and *aveBII* from the avermectin oleandrose (GenBank: AB032523.1) biosynthetic cluster (Supplementary Table 9). Avermectins are considered to be potent anthelmintics and insecticides (Wohlert *et al.* 2001), one example being the widely commercialized insecticide / acaricide / nematicide / anthelmintic abamectin from *Streptomyces avermitilis* (Khalil 2013). The results of this analysis indicated an array of possible secondary metabolites potentially produced by the two studied *Bacillus* strains. This information is useful for further experimental evaluation of these secondary metabolites for nematicidal activity, especially since the exact mechanism(s) of action by *B. firmus* against nematodes are not fully understood. Although the cuticle-degrading proteases are thought the primary virulence factor in *Bacillus* (Lian *et al.* 2007; Geng *et al.* 2016), it has been observed previously that various *B. firmus* strains elicited high nematode mortality rates, while having relatively few homologs or gene copies to known nematode-virulence factors in their genomes compared to other bacteria (Zheng *et al.* 2016). This could be due to high expression rates, novel interaction by known virulence factors, or the existence of as yet uncharacterized nematotoxic compounds.

### Conclusions

Through whole genome sequencing, comparative genomics and bioinformatics analysis it was possible to determine differences between the *B. firmus* I-1582 and *Bacillus* sp. ZZV12-4809 genomes and to assess their genetic capacity for nematode virulence. Although the *B. firmus* group appears to be relatively homogenous according to 16S rDNA, ANI analysis of whole genome assemblies available in GenBank highlighted previously unreported variability within the *B. firmus* group that probably mirrors the diverse habitats from which these strains were originally isolated. The differences in ANI% were sufficient to suggest incorrect species circumscription of some strains in GenBank. Various homologs to known nematode-virulent proteases from different organisms were found, specifically 18 and 19 different homologous sequences were found respectively in the *B. firmus* I-1582 and *Bacillus* sp. ZZV12-4809 strains. Additionally, two putative nematode-virulent chitinases were found in ZZV12-4809, containing the GH18-chitinase domain necessary to hydrolyze chitin and degrade chitinous structures in nematode eggs, thus contributing to their mortality. Additionally, 28 and 36 secondary metabolite clusters that could express an array of compounds belonging to siderophores, toxins, bacteriocins, lanthipeptides and antibiotics were predicted respectively in the genomes of *B. firmus* I-1582 and *Bacillus* sp. ZZV12-4809. The activity and nematotoxic potential of these putative secondary metabolites has yet to be experimentally characterized, but the resulting body of information points to a range of possible direct and indirect mechanisms of *B. firmus* and related species that could contribute to nematode virulence and could thus be used for targeted studies on virulence mechanisms, gene expression, and proteomic and metabolic studies of *B. firmus*, widely used as a biocontrol agent in crop production.
